# Association Study for 26 Candidate Loci in Idiopathic Pulmonary Fibrosis Patients from Four European Populations

**DOI:** 10.3389/fimmu.2016.00274

**Published:** 2016-07-11

**Authors:** Amit Kishore, Veronika Žižková, Lenka Kocourková, Jana Petrkova, Evangelos Bouros, Hilario Nunes, Vladimíra Loštáková, Joachim Müller-Quernheim, Gernot Zissel, Vitezslav Kolek, Demosthenes Bouros, Dominique Valeyre, Martin Petrek

**Affiliations:** ^1^Laboratory of Immunogenomics, Department of Pathological Physiology, Faculty of Medicine and Dentistry, Palacký University, Olomouc, Czech Republic; ^2^Laboratory of Pharmacology, University Hospital Alexandroupolis, Democritus University of Thrace, Athens, Greece; ^3^Université Paris 13, COMUE Sorbonne Paris Cité, Bobigny, Paris, France; ^4^Department of Respiratory Medicine, Faculty of Medicine and Dentistry, Palacký University, Olomouc, Czech Republic; ^5^Department of Pneumology, Center for Medicine, Medical Center, University of Freiburg, Freiburg, Germany; ^6^Academic Department of Pneumonology, Hospital for Diseases of the Chest ‘Sotiria’, Medical School, University of Athens, Athens, Greece; ^7^Faculty of Medicine and Dentistry, Institute of Molecular and Translational Medicine, Palacký University and Faculty Hospital, Olomouc, Czech Republic

**Keywords:** *MUC5B*, *MUC2*, cytokines, idiopathic pulmonary fibrosis, sequenom MassARRAY, single nucleotide polymorphism, association study, network analysis

## Abstract

Idiopathic pulmonary fibrosis (IPF) affects lung parenchyma with progressing fibrosis. In this study, we aimed to replicate *MUC5B* rs35705950 variants and determine new plausible candidate variants for IPF among four different European populations. We genotyped 26 IPF candidate loci in 165 IPF patients from four European countries, such as Czech Republic (*n* = 41), Germany (*n* = 33), Greece (*n* = 40), France (*n* = 51), and performed association study comparing observed variant distribution with that obtained in a genetically similar Czech healthy control population (*n* = 96) described in our earlier data report. A highly significant association for a promoter variant (rs35705950) of mucin encoding *MUC5B* gene was observed in all IPF populations, individually and combined [odds ratio (95% confidence interval); *p*-value as 5.23 (8.94–3.06); 1.80 × 10^−11^]. Another non-coding variant, rs7934606 in *MUC2* was significant among German patients [2.85 (5.05–1.60); 4.03 × 10^−4^] and combined European IPF cases [2.18 (3.16–1.50); 3.73 × 10^−5^]. The network analysis for these variants indicated gene–gene and gene–phenotype interactions in IPF and lung biology. With replication of *MUC5B* rs35705950 previously reported in U.S. populations of European descent and indicating other plausible polymorphic variants relevant for IPF, we provide additional reference information for future extended functional and population studies aimed, ideally with inclusion of clinical parameters, at identification of IPF genetic markers.

## Introduction

Idiopathic pulmonary fibrosis (IPF) is a chronic, progressive form of fibrosing interstitial pneumonia of unknown cause that predominantly affects lung parenchyma, leading to progressive worsening of dyspnea and lung function (1). In pathobiological mechanisms of IPF, role for gene variation has been implicated and spectrum of susceptible/protective polymorphic gene variants, including those in loci governing immune and inflammatory reactions and signaling processes, has been recently reported from genome-wide association studies (GWAS) or population-based case–control investigations (2–13); notably distribution of nominated gene variants varied among populations of different ancestry (Table S1 in Supplementary Material). Further, recent bioinformatics approaches yielded a genomic model that accurately predicted high- and low-risk IPF patients using a list of 118 IPF prognostic predictor genes, many of those with immune-, also T-cell-related functions (14). In aggregate, these reports implicated involvement of multiple genetic factors in IPF development and emphasized the need for their evaluation in different populations to decipher the plausible pathobiological mechanism of IPF.

In context of the above efforts, we have recently identified and reported 26 IPF-associated candidate loci (15). Besides characterizing their major functions, e.g., in regulating production of mucins (*MUC5B* and *MUC2*) or of pro-inflammatory cytokines (IL-1, IL-8) and also in cell signaling and innate immunity processes (*TLR3* and *TOLLIP*) involved in inflammatory and profibrotic pathways (http://www.ncbi.nlm.nih.gov/pmc/articles/PMC4585032/table/T1/), we have described the approach for their simultaneous investigation using a novel mass spectrometry based matrix-assisted laser desorption/ionization time-of-flight (MALDI-TOF) multiplexed genotyping assay and reported their gene frequencies in healthy Czech (European) population (15). There, we also suggested the wider application of the data from this report for association studies among genetically homogenous populations following the recommendation of STrengthening the REporting of Genetic Association studies (STREGA) (16). Suggestion to include this particular control population in the present IPF association study in different European populations was based on reports of genetic similarity among Europeans, for example from results of genotyping 6000 individuals as control samples for >300,000 single nucleotide polymorphisms (SNPs) in a GWAS (17) and findings of a HapMap study (18). Further, in our decision we also reflected recommendations of HLA-NET network group for usage of geographical and/or cultural criteria to describe human populations (19) and also our own observations of substantial degree of homogeneity in distribution of immune-related gene variants within European populations, including Greeks (20).

Hereby, we report the results from a multicenter association gene study in which we determined the status (genotype distribution, genotype, and allele frequencies with carriage rate) of 26 IPF candidate loci. Here, we have performed a comparative study for association of these IPF candidate genetic variants among four different European (Czech, German, Greek, and French) populations and have ammended it by network prediction for gene–gene/gene–phenotype interactions in IPF and lung biology. We suggest that future extended and replicative studies following hereby described approach could enable better understanding of IPF pathogenesis, and if further supported by patient laboratory and clinical data, it could help to nominate novel disease markers.

## Materials and Methods

### Characteristics of IPF Cases

In this study, 165 IPF patients from four European populations comprising 41 Czechs (Centre: University Hospital, Olomouc), 33 Germans (University Medical Center, Freiburg), 40 Greeks (Medical School University, Athens), and 51 French (University Hospital, Paris), representing Central, Southern, and Western Europe, were enrolled (Table 1).

**Table 1:** **Subjects characteristics under study comprising IPF cases from four different populations**.

	Control subjects^a^	European IPF cases				
		Czech	German	Greek	French	Total IPF
*N*	96	41	33	40	51	165
Age, mean ± SD	34.45 ± 8.94	59.78 ± 10.12	66.25 ± 12.28	72.15 ± 7.71	72.19 ± 11.97	67.97 ± 11.60
Age, range	18–57	42–81	36–85	51–88	36–92	36–92
Males:females	45:51	23:18	26:7	32:8	44:7	125:40

*^a^Healthy control subjects from our previous data report (15)*.

All subjects were unrelated, white, and of European origin living in specified countries of Europe and speaking their respective national languages. The IPF cases were diagnosed as per ATS/ERS/JRS/ALAT guidelines (1, 21, 22) with typical clinical features and abnormalities on chest high-resolution computed tomography (HRCT) scans, abnormal lung function tests with reduced diffusing capacity of the lung for CO (DL_CO_), and/or restrictive pulmonary deficit, exclusion of other known causes of interstitial lung disease (ILD). For comparisons of genotype, allele frequency, and carriage rate (phenotype frequency) of analyzed genomic variants and case–control association study, we have utilized the data on distribution of these variants in 96 Czech healthy controls (15). Genomic DNA was isolated from peripheral blood leukocytes by standard salting out method (23). Informed consent was obtained from all study participants. The study was performed with approval of institutional ethical committees at respective centers (Ethics Commitee of University Hospital and Medical Faculty of Palacky University, Olomouc, Czech Republic; Ethics Committee of the University Hospital Freiburg, Germany; Ethics Committee Hospital for Diseases of the Chest, Athens, Greece; and Comité Consultatif de protection des Personnes dans la recherche biomédicale-hôpital Robert Ballanger, France).

### Assay Design, PCR Amplification and Genotyping

The details of the panel comprising 26 IPF candidate loci and genotyping procedure in IPF cases have been described previously (15). In brief, a total of 26 SNPs reported as associated with IPF in literature were selected. These SNPs were located within the genes of different functional categories (mucus production, pro-inflammatory cytokines, chemokines, innate immune response, telomerase maintenance, cell surface remodeling, GTPase activator activity, cell-cycle regulators, phospholipid translocators, desmoplakin production, etc.). For PCR amplification and single base extension (SBE) reaction, the primer pairs along with extension primers were designed using Assay design suite v2.0. These primers were multiplexed and genotyped using Sequenom MassARRAY platform integrating iPLEX^®^ SBE reaction and MassARRAY^®^ technology (Agena Bioscience, San Diego, CA, USA) based MALDI-TOF MS assay. The assay consists of an initial locus-specific PCR amplification followed by SBE using mass-modified dideoxynucleotide terminators of an oligonucleotide primer that anneals immediately upstream of the target polymorphic site. The distinct mass of extended primer traces the alternative alleles using MassARRAY Typer 4.0.20. For quality control (QC) step, we determined data missing rate per individuals and missing rate per SNP. Also, for QC of SNP genotyping, positive and negative template control samples were included in each assay plate. Any assay found as positive in negative template control were removed from the study.

### Statistical Analysis

Each SNP was tested for Hardy–Weinberg equilibrium (HWE) by Pearson’s Chi-square (χ^2^) test or Fisher exact test, as applicable. SNPs within HWE (*p* > 0.05) and sufficiently common [minor allele frequency (MAF) > 5%] in studied population were included. Carriage rate (phenotype frequency) was calculated as number of individuals carrying one (or two) copies of a particular allele on one or both (maternal and paternal) chromosomes. Association of SNPs minor alleles with IPF susceptibility were evaluated by Fisher’s exact test providing odds ratio (OR), 95% confidence interval (CI), and level of significance (*p*). For Bonferroni correction of multiple comparison (number of test = 100; 20 SNPs for four individual and the combined populations), a stringent approach with *p*-value < 0.05/100 (5 × 10^−4^) was considered as significant.

### Network Analysis

Prediction of gene–gene network for plausible candidate variants (*p* < 0.05) and their interaction with IPF and other phenotypes, such as lung disease, lung injury, and lung function was performed using Phenolyzer, a tool for phenotype-based prioritization of candidate genes in human diseases (24). The candidate genes and their relationship with IPF and related phenotypes were investigated in several databases to determine and score relevant seed genes. The seed genes are then expanded to include related genes, on the basis of several types of gene–gene relationship components, such as exhibiting a protein–protein interaction, sharing a gene family or biological pathway, or transcriptionally regulating or being regulated by another gene. Finally, these different types of scores from seed gene ranking and gene–gene relationships are integrated to generate a ranked candidate gene list, together with gene–gene and gene–phenotypes interactions used to normalize the scores in range 0–1 (24).

## Results

The characteristics of IPF patients and healthy control subjects included in this study are presented in Table 1. The proportions of IPF male cases were higher than of female cases. Following QC steps for missing rates, the genotyping data from all individual passed the QC with (i) missing frequency per individual 0.038 (N_MISS = 1) to 0.077 (N_MISS = 2) (a single IPF case from Greek population that failed the assay was not included in this study), and (ii) genotype missing frequency (F_MISS/assay error rate; Table 2) 0.004 to 0.015. Five SNPs showed departure from HWE (Table S2 in Supplementary Material), namely, *PRKCE* rs628877 (*p* = 0.02 in combined IPF cases with F_MISS = 0.008), *IL-4* rs2243250 (*p* = 0.01, F_MISS = 0.0146 in Czech IPF and *p* = 1.2 × 10^−4^, F_MISS = 0.011 in combined IPF cases), *IL-4* rs2070874 (*p* = 0.02, F_MISS = 0 in Czech IPF), *IL-4R*α rs1801275 (*p* = 0.04, F_MISS = 0 in Czech healthy controls; *p* = 0.03, F_MISS = 0 in Greek IPF and *p* = 0.01, F_MISS = 0 in combined IPF cases), and *MAPT* rs1981997 (*p* = 0.03, F_MISS = 0.007 in French IPF cases). Further, with MAF threshold check, *TP53* rs12951053 was found with MAF = 0.04, F_MISS = 0 in Greek IPF cases. Thus, keeping the QC stringent conditions, these six SNPs were removed from further analysis, for which 20 variants remained (Table 2).

**Table 2:** **Allelic model of association for IPF risk among four European populations**.

S. No.	Gene	SNP	Minor allele	Czechs	Germans	Greeks	French	Total	Assay error rate
				OR (95% CI) *p*	OR (95% CI) *p*	OR (95% CI) *p*	OR (95% CI) *p*	OR (95% CI) *p*	
1	*IL-1*α	rs1800587	T	0.65 (1.16–0.37) 0.16	0.78 (1.42–0.42) 0.45	0.56 (1.01–0.31) 0.06	0.74 (1.25–0.44) 0.30	0.68 (0.99–0.46) 4.94 × 10^−2^	0
2	*L-1*β	rs16944	A	1.15 (1.98–0.67) 0.67	0.79 (1.46–0.42) 0.54	1.01 (1.76–0.58) 1.00	1.14 (1.9–0.69) 0.61	1.03 (1.51–0.71) 0.92	0
3	*IL-1*β	rs1143634	T	1.06 (1.93–0.59) 0.88	1.34 (2.49–0.72) 0.42	0.71 (1.37–0.37) 0.34	1.06 (1.84–0.61) 0.89	1.02 (1.54–0.68) 1.00	0
4	*LRRC34*	rs6793295	C	0.87 (1.55–0.49) 0.66	0.96 (1.77–0.52) 1.00	0.79 (1.43–0.44) 0.46	1.35 (2.24–0.81) 0.29	1.00 (1.48–0.68) 1.00	0
5	*TF*	rs1799899	A	1.46 (3.47–0.61) 0.48	2.89 (6.47–1.3) 1.12 × 10^−2^	1.88 (4.3–0.82) 0.17	2.20 (4.65–1.04) 4.58 × 10^−2^	2.06 (3.78–1.12) 1.87 × 10^−2^	0
6	*IL-8*	rs4073	A	1.28 (2.14–0.76) 0.36	1.16 (2.03–0.66) 0.67	1.16 (1.95–0.69) 0.60	1.11 (1.8–0.69) 0.71	1.17 (1.67–0.82) 0.41	0
7	*FAM13A*	rs2609255	G	1.01 (1.82–0.56) 1.00	0.79 (1.54–0.4) 0.51	1.04 (1.88–0.57) 0.88	1.46 (2.47–0.86) 0.17	1.09 (1.64–0.73) 0.68	0
8	*TLR3*	rs3775291	A	0.94 (1.62–0.54) 0.89	0.61 (1.16–0.32) 0.17	0.72 (1.29–0.41) 0.32	0.72 (1.22–0.43) 0.24	0.75 (1.1–0.51) 0.14	0
9	*TERT*	rs2736100	G	1.01 (1.70–0.60) 1.00	0.85 (1.51–0.48) 0.67	0.49 (0.83–0.29) 1.12 × 10^−2^	1.23 (1.99–0.76) 0.46	1.23 (1.76–0.86) 0.28	0
10	*IL-13*	rs1800925	T	0.66 (1.22–0.35) 0.23	0.65 (1.27–0.34) 0.26	1.08 (1.91–0.61) 0.88	0.83 (1.43–0.48) 0.59	0.80 (1.2–0.54) 0.30	0.008
11	*IL-4*	rs2243248	G	0.47 (1.69–0.13) 0.29	0.59 (2.13–0.16) 0.57	1.78 (4.19–0.75) 0.24	1.20 (2.88–0.5) 0.66	1.02 (2.01–0.52) 1.00	0.008
12	*CDNK1A*	rs733590	C	1.21 (2.05–0.72) 0.50	0.76 (1.38–0.42) 0.46	1.15 (1.95–0.67) 0.68	1.01 (1.65–0.62) 1.00	1.03 (1.49–0.72) 0.93	0
13	*OBFC1*	rs11191865	A	1.67 (2.81–0.99) 0.06	1.75 (3.07–0.99) 0.06	1.07 (1.8–0.63) 0.89	1.43 (2.31–0.88) 0.18	1.44 (2.06–1.01) 0.06	0
14	*MUC2*	rs7934606	A	1.41 (2.41–0.83) 0.21	**2.85 (5.05–1.60) 4.03 × 10^−4^**	2.45 (4.19–1.43) 1.43 × 10^−3^	2.36 (3.86–1.44) 7.03 × 10^−4^	**2.18 (3.16–1.50) 3.73 × 10^−5^**	0.004
15	*MUC5B*	rs35705950	T	**3.77 (7.47–1.9) 1.62 × 10^−4^**	**4.83 (9.79–2.39) 1.55 × 10^−5^**	**5.46 (10.82–2.76) 1.13 × 10^−6^**	**6.77 (12.65–3.62) 5.28 × 10^−10^**	**5.23 (8.94–3.06) 1.80 × 10^−11^**	0.015
16	*ATP11A*	rs1278769	A	1.10 (1.92–0.63) 0.77	0.53 (1.06–0.26) 0.08	1.02 (1.79–0.57) 1.00	0.51 (0.92–0.28) 2.51 × 10^−2^	0.76 (1.13–0.51) 0.18	0
17	*TP53*	rs12602273	G	2.43 (5.3–1.11) 3.10 × 10^−2^	1.40 (3.6–0.54) 0.45	1.13 (2.89–0.44) 0.81	1.57 (3.5–0.71) 0.29	1.63 (3.03–0.87) 0.14	0
18	*ACE*	rs4277405	C	0.54 (0.96–0.30) 3.71 × 10^−2^	1.08 (1.92–0.61) 0.88	1.11 (1.9–0.65) 0.78	1.12 (1.83–0.68) 0.71	0.94 (1.36–0.65) 0.78	0
19	*ACE*	rs4459609	C	0.54 (0.96–0.30) 3.71 × 10^−2^	1.08 (1.92–0.61) 0.88	1.11 (1.9–0.65) 0.78	1.08 (1.76–0.66) 0.80	0.93 (1.34–0.64) 0.71	0
20	*DPP9*	rs12610495	G	1.01 (1.82–0.56) 1.00	1.56 (2.85–0.86) 0.15	1.01 (1.84–0.55) 1.00	1.14 (1.96–0.66) 0.68	1.15 (1.72–0.77) 0.54	0.008

*Bold values are significant with Bonferroni corrected p < 5 × 10^−4^*.

*OR: Odds ratio; CI: Confidence interval*.

The primary analysis (*p* < 0.05) using allelic (multiplicative) genetic model revealed a total of nine SNPs for IPF susceptibility. Among these, three SNPs were shared among different IPF populations: first, rs35705950*T within promoter region of *Mucin5B* (*MUC5B*) was highly significant among all the IPF populations – Czech [OR (95% CI); *p*: 3.77 (7.47–1.9); 1.62 × 10^−4^]; German [4.83 (9.79–2.39); 1.55 × 10^−5^]; Greek [5.46 (10.82–2.76); 1.13 × 10^−6^]; French [6.77 (12.65–3.62); 5.28 × 10^−10^]; and combined IPF cases [5.23 (8.94–3.06); 1.80 × 10^−11^] (Table 2). Second, rs7934606*A within intron region of *Mucin2* (*MUC2*) was significant for German [2.85 (5.05–1.60); 4.03 × 10^−4^], Greek [2.45 (4.19–1.43); 1.43 × 10^−3^], French [2.36 (3.86–1.44); 7.03 × 10^−4^], and combined IPF [2.18 (3.16–1.50); 3.73 × 10^−5^] cases. The third variant, rs1799899*A located in exon region of *Transferrin* (*TF*) gene was significant for Germans [2.89 (6.47–1.30); 1.12 × 10^−2^], French [2.20 (4.65–1.04); 4.58 × 10^−2^], and in combined IPF [2.06 (3.78–1.12); 1.87 × 10^−2^] cases. Prediction of loss-/gain-of-function for functional variant *TF* rs1799899 suggested it as a probably damaging mutation (Polyphen score: 0.869 and SIFT score: 0.07).

The other six significant SNPs were featured in individual populations of which three were associated with IPF in Czech: (i) rs12602273*G in *Tumor protein 53* (*TP53*) [2.43 (5.3–1.11); 3.10 × 10^−2^]; (ii) rs4277405*C in *Angiotensin converting enzyme* (*ACE*) [0.54 (0.96–0.30); 3.71 × 10^−2^]; and (iii) rs4459609*C in *ACE* [0.54 (0.96–0.30); 3.71 × 10^−2^] and one each in Greek: *Telomerase reverse transcriptase* (*TERT*) rs2736100*G [0.49 (0.83–0.29); 1.12 × 10^−2^]; French: *ATPase, type 11A* (*ATP11A*) rs1278769*A [0.51 (0.92–0.28); 2.51 × 10^−2^]; and in total IPF cases: *Interleukin-1* α (*IL-1*α) rs1800587*T [0.68 (0.99–0.46); 4.94 × 10^−2^] (Table 2).

These findings of allelic model were in concordance with Pearson’s χ^2^ test and additive test of logistic regression analysis with similar panel and population distribution/stratification of significant risk variants, and an exceptional addition of rs11191865*A (*p* = 0.046) in *Oligonucleotide/oligosaccharide-binding fold containing 1* (*OBFC1*) exclusively in total IPF set under χ^2^ test.

**Figure 1:**
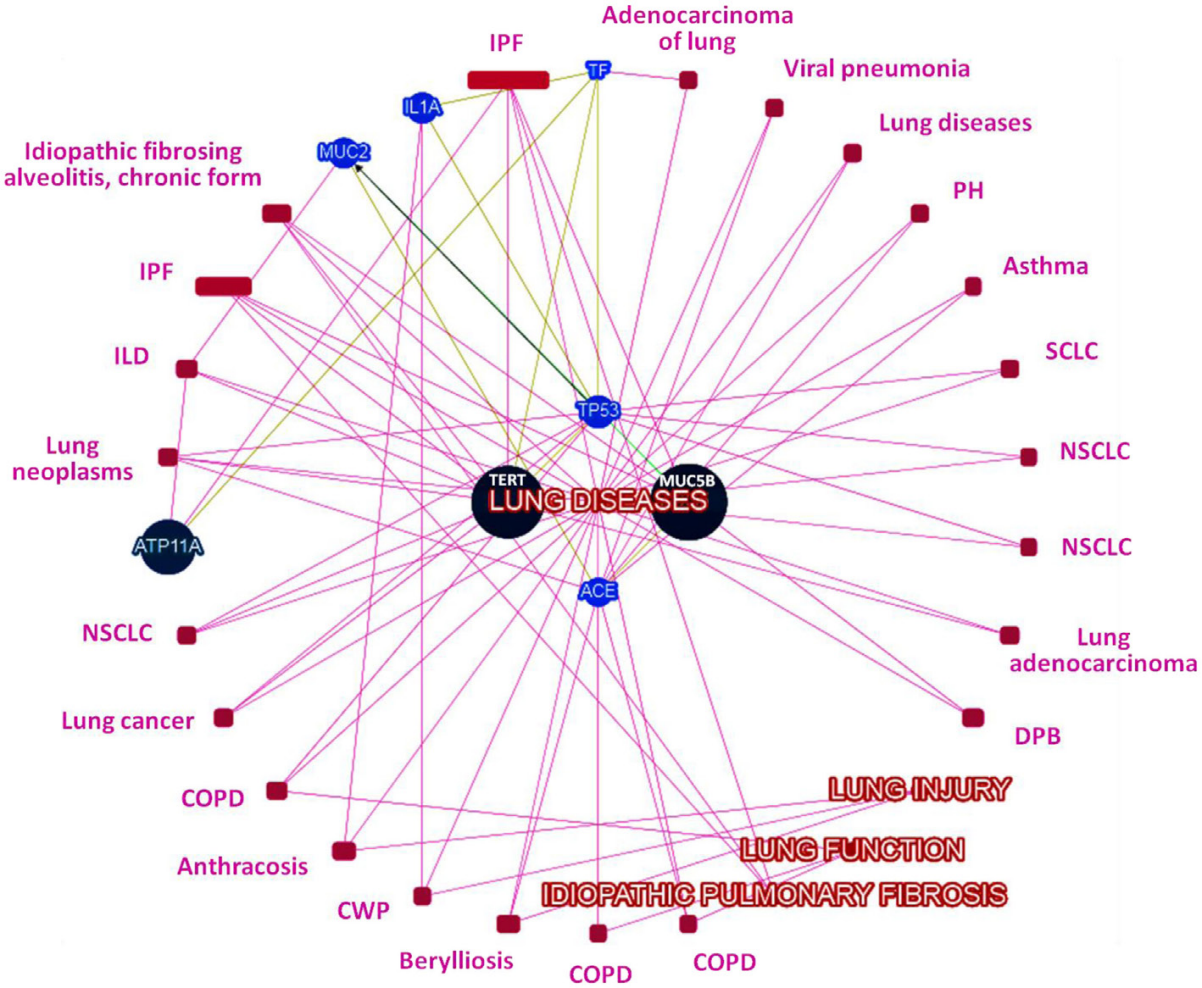
**Network of genes with candidate loci and their interaction with IPF, lung injury, lung function, and lung disease phenotypes (ILD, interstitial lung disease; CWP, coal worker’s pneumoconiosis; COPD, chronic obstructive pulmonary disease; DPB, diffuse panbronchiolitis; NSCLC, non-small cell lung cancer; SCLC, small cell lung cancer; PH, pulmonary hypertension)**.

**Figure 2:**
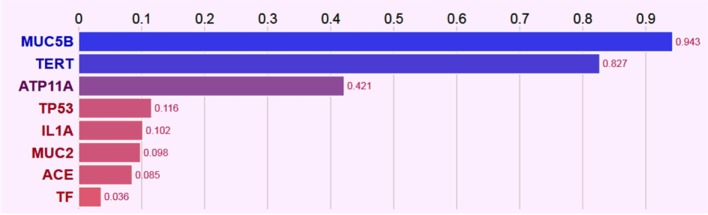
**Bar plot of seed genes (*MUC5B, TERT*, and *ATP11a*) and predicted genes (*TP53, IL1A, MUC2, ACE*, and *TF*) normalized scores based on the gene–gene and gene–phenotype interactions**.

Network analysis among genes with significant variants showed *MUC5B* and *MUC2* in same gene family (Figure S1 in Supplementary Material), transcriptional interaction among *MUC2* and *TP53* (Figure S2 in Supplementary Material), and all identified candidate genes belonged to the same biosystem (Figure S3 in Supplementary Material). Further prediction based on their records in several databases (CLINVAR, ORPHANET, OMIM, DISGENET, GWAS, HTRI, GENE_FAMILY, and BIOSYSTEM), gene–gene interaction and interaction with phenotypes (IPF, lung disease, lung injury, and lung function) has (Figure 1) prioritized *MUC5B* as highest-ranked genes and indicated *MUC5B, TERT*, and *ATP11a* as seed genes, while *TP53, IL1A, MUC2, ACE*, and *TF* as predictive genes based on their normalized scores (Figure 2).

Applying rather conservative Bonferroni correction for multiple comparisons (100 tests, see Materials and Methods), *MUC5B* rs35705950*T remained as highly significant in all IPF populations, Czech (*p* = 1.62 × 10^−4^), German (1.55 × 10^−5^), Greek (1.13 × 10^−6^), French (5.28 × 10^−10^), and in combined IPF population (1.80 × 10^−11^); whereas *MUC2* rs7934606*A was significant in German (4.03 × 10^−4^) and in combined IPF (3.73 × 10^−5^) (Table 2). The inheritance hypothesis for Bonferroni significant variants, *MUC5B* rs35705950 and *MUC2* rs7934606, under allelic model for IPF association were also tested for other models and the variant rs35705950 was found in concordance to the dominant model indicating allele T for increased risk (Table 3).

**Table 3:** **Dominant model of inheritance for highly significant IPF susceptible risk variants with Bonferroni correction among the four European populations**.

Genetic variants	Czech	German	Greek	French	Total IPF
	OR (95% CI); *p*	OR (95% CI); *p*	OR (95% CI); *p*	OR (95% CI); *p*	OR (95% CI); *p*
*MUC5B* rs35705950*T (GT + TT vs. GG)	**4.76 (2.11–10.75); 2.21 × 10^−4^**	**8.75 (3.59–21.30); 9.04 × 10^−7^**	**6.25 (2.67–14.60); 1.97 × 10^−5^**	**9.167 (4.18–20.12); 9.6 × 10^−9^**	**7.01 (3.77–13.06); 6.21 × 10^−11^**
*MUC2* rs7934606*A (GA + AA vs. GG)	1.29 (0.61–2.74); NS	**7.45 (2.13–26.12); 2.73 × 10^−4^**	2.48 (1.06–5.80); 4.83 × 10^−2^	2.71 (1.24–5.92); 1.16 × 10^−2^	2.47 (1.43–4.26); 1.27 × 10^−3^

*Bold values are significant with Bonferroni corrected p < 5 × 10^−4^*.

*OR, Odds ratio; CI, Confidence interval; NS, Non-significant*.

For the highly significant variants, we also report the allele frequency of rs35705950*T risk allele to range from 0.09 to 0.41 and rs7934606*A from 0.32 to 0.58 among the analyzed four European populations (Table S2 in Supplementary Material). Among IPF cases, the allele frequency and carriage rate for rs35705950*T were lowest in Czech (0.28 and 0.49, respectively) and highest in French (0.41 and 0.65, respectively); while rs7934606*A has lowest frequencies in Czech (0.40 and 0.63, respectively) and highest in Germans (0.58 and 0.91, respectively).

## Discussion

This study is a first report of comparative distribution of genotypes and alleles of 26 candidate gene variants implicated in mucin production, cell-cycle regulation, pro-inflammatory, and profibrotic signaling pathways pertinent to IPF pathobiology among patients from four populations across Europe (Czech, German, Greek, and French). Employing stringent statistical approach (*p* < 5 × 10^−4^), we report a high association between *MUC5B* rs35705950*T and IPF susceptibility in all the four analyzed populations among Czechs: [OR (95% CI) *p*] 3.77 (7.47–1.9) 1.62 × 10^−4^, Germans: 4.83 (9.79–2.39) 1.55 × 10^−5^, Greeks: 5.46 (10.82–2.76) 1.13 × 10^−6^, and French: 6.77 (12.65–3.62) 5.28 × 10^−10^ along with all patients from this study as a whole: 5.23 (8.94–3.06) 1.80 × 10^−11^. Thus, we replicate previous findings and provide an insight for predominant association of gel-forming mucin-encoding gene variants with IPF in Europeans and populations of European descent. The role of mucin variants was also supported with high significance of *MUC2* rs7934606 among Germans [2.85 (5.05–1.60); 4.03 × 10^−4^] and combined European IPF cases [2.18 (3.16–1.50); 3.73 × 10^−5^]. The identified nine variants among *MUC5B, MUC2, TF, TP53, ACE, TERT, ATP11A*, and *IL-1*α (significant at *p* < 0.05) were interactive at gene–gene and gene–phenotype level in network analysis. The present study for inter-population comparison is imperative with respect of comparing the distributions of plausible IPF-associated gene variants and, thus, may provide starting point(s) for further investigations of biological (functional implication of nominated loci) and translational (relationship with laboratory/clinical parameters) aspects.

Among mucin gene cluster (*MUC6, MUC2, MUC5AC*, and *MUC5B*) on chromosome 11q15.5, *MUC5B* is the most predominant in the normal distal airway epithelium and is widely associated with sporadic IPF and familial interstitial pneumonia (9, 25–27). The *MUC5B* promoter-variant rs35705950 SNP predicted change in transcription factors binding sites (disruption of E2F and creation of HOX9 and PAX2) and the risk T-allele has been strongly associated with increased *MUC5B* expression in the lung tissue. The relative high frequency of rs35705950*T risk allele (IPF cases: 0.28–0.41 and healthy controls: 0.09) observed in this study are in concordance with several reports, including populations of European descent (Table S1 in Supplementary Material). To explain the mucin-expressing structures in IPF pathogenesis, Seibold et al. analyzed ciliated, basal, and alveolar type II cells in lung tissue and reported predominant expression of *MUC5B* in pseudostratified mucociliary epithelium comprised of basal epithelial cells and mucus cells in distal airway. Further, it is suggested that mucociliary dysfunction in the distal airway causing honeycomb cyst may play a role in the development of progressive fibroproliferative lung disease (25, 27). Although rs35705950 is reported as highly associated with IPF in Europeans and in populations of European descent, interestingly, it is weakly associated in East-Asians, such as Chinese, Japanese, and Koreans (Table S1 in Supplementary Material). Another mutation in the same gene family (Figure S1 in Supplementary Material) identified *MUC2* rs79834606 as significantly associated in IPF (*p* = 3.8 × 10^−6^) with MAF (A-allele) 0.54 in IPF cases and 0.41 in controls (9), which is in accordance to our present findings (IPF: 0.40–0.58 and controls: 0.32) (Table S2 in Supplementary Material). Similarly, MAF of 0.52 for rs79834606*A was reported among IPF cases in a GWAS (3).

Here, we newly identified rs1799899 located in the *TF* gene as IPF risk variant in Germans, French, and in combined European IPF cases, significant at primary analysis (0.05 > *p* > 5 × 10^−4^). The SNP rs1799899 marks a Gly/Ser change located in *TF* gene encoding a glycoprotein involved in iron ion transport and removal of certain organic matter and allergens from serum. As this is the first implication of *TF* gene in context of IPF, in parallel to the studies of its possible functional role, this association requires replication (16). The other six IPF risk variants identified in this study were featured among individual populations (Table 2). Among Czech IPF cases, (i) SNP rs12602273 located in intron of *TP53* that regulates cell-cycle arrest; while, (ii) rs4277405 and (iii) rs4459609 located in promoter region of *ACE-II* suggested with critical profibrotic role in IPF (28, 29) were observed. The MAF of rs12602273*G reported as 0.07 in IPF case and 0.08 in healthy population of the Netherlands (11) was comparable to our current findings (IPF: 0.09–0.12 and controls: 0.08). For Greek IPF cases, (iv) a common variant rs2736100 within intron of *TERT* and (v) rs1278769 in 3′-UTR of *ATP11A* were significant. Several studies have reported the telomerase gene mutation causing short telomerase as risk factor and poor survival in IPF (2, 30, 31). These variants, rs2736100*G and rs1278769*A, were initially reported in a GWAS study for IPF cases with MAF of 0.43 and 0.20, respectively (MAF = 0.41–0.63 and 0.18–0.30, respectively in this study) (3). Besides, (vi) rs1800587 in 5′-flanking region of pro-inflammatory cytokine *IL-1*α was lesser significant among the combined IPF cases. Earlier, a study in Czech population has reported rs1800587*T MAF of 0.30 in IPF cases and 0.32 in healthy controls (4), similar to our current finding (IPF: 0.27–0.29 and controls: 0.36).

While *MUC5B* rs35705950 is highly replicable, the other probable candidate variants (including new report of *TF* rs1799899 in IPF) reported in this study must be replicated in accordance to the guidelines for conductance of genetic association studies (16), by other independent studies. However, already at this stage, our analysis of the gene–gene and gene–phenotype interaction networks suggests that these candidate variant genes are pertinent to IPF and lung function biology (Figure 1) and provides support for biological plausibility of observed variants.

Apart from using Czech population control data for comparisons within the European context, which was noted and reasoned for in the Section “Introduction,” another limitation of this study is that a single national center was included for each of four European populations, where a relatively small number of IPF cases were genotyped, which reduces the power of our present findings. However, with a conservative incidence range of 3–9 cases per 100,000 per year for Europe (32), the enrolled sample size (165 IPF cases) in the present study considerably represents the disease among Europeans; moreover, it is comparable with sizes of other reported studies (Table S1 in Supplementary Material). Off note, our primary aim was to report our findings of the candidate IPF genetic variants so that these could be replicated in other centers (16) and investigated further. In this regard, investigations of relationships with clinical parameters such as lung functions will follow.

Our findings provide evidence that gene variants involved in mucin production (*MUC5B* and *MUC2*) do increase IPF risk among the four European populations, two of which (Czech and Greek) have not been studied before in this regard at all. Additionally, the nominated variants in *TF* and other variants of *TP53, ACE, TERT, ATP11A*, and *IL-1*α may also contribute to IPF susceptibility. Despite our panel of 26 gene variants was designed across pertinent pathobiological pathways, it did not include others from the wide range of plausible IPF-associated SNPs regulating immune and fibrotic functions, such as master regulator TGF-β, TNF-α, full spectrum of TLRs, MHC (HLA) variants, and also SNPs in regulatory microRNAs (miRNAs) (4, 8, 33, 34). Therefore, these variants should be prioritized in the future studies aimed at extending a profile general and population-specific IPF gene biomarkers, including exploration of their functions, so that our view on the role of gene variation in origin of IPF and its further development more closely approaches the reality.

## Conclusion

The present study confirms and further extends strong association of *MUC5B* promoter region variant (rs35705950) with IPF disease among Europeans. In addition, it suggests further IPF-associated polymorphisms: *MUC2* (rs79834606) and *TF* (rs1799899) variants in general, and the other six (*TP53* rs12602273, *ACE-II* rs4277405, *ACE-II* rs4459609, *TERT* rs2736100, *ATP11A* rs1278769, and *IL-1*α rs1800587) in individual European populations. Their further investigation for disease association among extended patient cohorts is, therefore, warranted. At the same time, findings of our present study represent reference information to be utilized for future extended functional and population studies as well as for translational research of the nominated variants aiming at characterization of biomarkers and/or novel therapeutic targets.

## Ethics Statement

The study was approved by the ethical committee of the University Hospital, Olomouc; University Medical Center, Freiburg; Medical School University, Athens; and University Hospital, Paris. After approval of the study by ethical committee, the consent of human participants visiting the centres was taken in written for usage of biological sample for research purpose in future.

## Author Contributions

MP, GZ, JM-Q, JP, VK, DB, and DV conceived and designed the work; AK, VZ, LK, JP, EB, HN, VL, GZ, VK, and MP contributed to data acquisition; AK performed data analysis and interpretation; AK and MP drafted the manuscript after its revision for important intellectual context by all authors; MP and AK finalized the article. All authors have read and approved the final manuscript and agreed to be accountable for all aspects of the work.

## Conflict of Interest Statement

The authors declare that the research was conducted in the absence of any commercial or financial relationships that could be construed as a potential conflict of interest.
