# Supplementary material for: Association Study for 26 Candidate Loci in Idiopathic Pulmonary Fibrosis Patients from Four European Populations
Source: Front Immunol. 2016 Jul 11;7:274. doi: 10.3389/fimmu.2016.00274 (PMC4939450; doi:10.3389/fimmu.2016.00274)
Supplement: Supplementary file 1 [file Image_1.DOCX]

***Supplementary Material***

**Association Study for 26 Candidate Loci in Idiopathic Pulmonary Fibrosis Patients from Four European Populations**

**Amit Kishore^1^, Veronika Žižková^1^, Lenka Kocourková^1^, Jana Petrkova^1^, Evangelos Bouros^2^, Hilario Nunes^3^, Vladimíra Loštáková^4^, Joachim Müller-Quernheim^5^, Gernot Zissel^5^, Vitezslav Kolek^4^, Demosthenes Bouros^6^, Dominique Valeyre^3^, Martin Petrek ^1,7^***

^1^Department of Pathological Physiology, Laboratory of Immunogenomics, Faculty of Medicine and Dentistry, Palacký University, Olomouc, Czech Republic

^2^Pharmacology Lab, Democritus University of Thrace and University Hospital Alexandroupolis, Athens, Greece

^3^Université Paris 13, COMUE Sorbonne Paris Cité, Bobigny - Paris, France

^4^Department of Respiratory Medicine, Faculty of Medicine and Dentistry, Palacký University, Olomouc, Czech Republic

^5^Department of Pneumology, Center for Medicine, Medical Center, University of Freiburg, Freiburg, Germany

^6^Academic Department of Pneumonology, Medical School, University of Athens, Hospital for Diseases of the Chest 'Sotiria' - Athens, Greece

^7^Institute of Molecular and Translational Medicine, Faculty of Medicine and Dentistry, Palacký University and Faculty Hospital, Olomouc, Czech Republic

***Correspondence:** dr. M. Petrek, Department of Pathological Physiology, Faculty of Medicine and Dentistry, Palacky University, Olomouc, Hněvotínská str. 3, 77515, Czech Republic; E-mail: martin.petrek@fnol.cz

**Supplementary Tables**

**Table S1:** Studies reporting *MUC5B* rs35705950*T allelic association with idiopathic pulmonary fibrosis (IPF).

**Table S2:** Genotype distribution of 26 variants studied, their genotype/ allele frequencies and carriage rate in Czech healthy controls (HC*), IPF cases from four European (CZ: Czechs, GE: Germans, GR: Greek and FR: French) and the combined IPF populations.

**Table S1:** Studies reporting *MUC5B* rs35705950*T allelic association with idiopathic pulmonary fibrosis (IPF).

| **S.No.** | **Study** | **Ancestry (Population)** | **IPF cases** | **Controls** | **MAF** | | | **OR (95% CI); p-value** | | **Reference** | |
| --- | --- | --- | --- | --- | --- | --- | --- | --- | --- | --- | --- |
|  |  |  |  |  | **Case (%)** | **Control (%)** |  | |  | |  |
| 1 | Candidate | European-Americans | 492 | 322 | 37.5 | 9.1 | 5.95 (4.42-8.01); <1.00x10^-8^ | | (1) | |  |
| 2 | Candidate | European-Americans (NHW individuals) | 47 | 1370 | 23.4 | 9.8 | 6.3 (3.1-12.7); <0.001 | | (2) | |  |
| 3 | Candidate | Hispanic (Mexican) | 83 | 111 | 25.0 | NR | 7.36 (NR); 1.1x10^-4^ | | (3) | |  |
|  |  | East-Asians (Korean) | 239 | 87 | 1.0 | 0 | Non significant | | (3) | |  |
| 4 | Candidate | European-Americans (NHW of US) Pittsburgh | 246 | 166 | 33.7 | 10.8 | 4.19 (2.83-6.20); 9.50x10^-13^ | | (4) | |  |
|  |  | European-Americans (NHW of US) Chicago | 95 | 636 | 35.8 | 11.2 | 4.44 (3.14-6.26); 1.75x10^-19^ | | (4) | |  |
|  |  | European-Americans (NHW of US) Combined | 341 | 802 | 34.3 | 11.1 | 4.18 (3.35-5.22): 7.56x10^-40^ | | (4) | |  |
| 5 | Candidate | East-Asians (Japanese) | 44 | 310 | 3.4 | 0.8 | 4.34 (1.02-18.49); 0.047 | | (5) | |  |
|  |  | Europeans (Germans) | 71 | 35 | 33.1 | 4.3 | 11.05 (3.3-36.99); 9.75x10^-5^ | | (5) | |  |
| 6 | Candidate | East-Asians (Chinese) | 165 | 1013 | 3.3 | 0.8 | 4.33 (1.99-9.42); 2.16x10^-4^ | | (6) | |  |
| 7 | Candidate | European-Americans | 84 | 689 | 29.8 | 11.7 | 3.20 (2.21-4.63); 1.2x10^-10^ | | (7) | |  |
| 8 | Candidate | European (Netherlands) | 115 | 249 | 26.5 | 9.0 | 3.63 (2.38-5.55); 3.3x10-9 | | (8) | |  |
| 9 | Candidate | Europeans (French) | 142 | 1383 | 38.7 | 10.8 | 5.22 (3.99-6.81); <1.00x10^-8^ | | (9) | |  |
| 10 | Candidate | Europeans (UK) | 110 | 416 | 35.9 | 10.0 | 5.06 (3.54-7.22); <1.00x10^-8^ | | (10) | |  |
| 11 | GWAS (Replication) | European-Americans | 1616 (876) | 4683 (1890) | NR | NR | 4.51 (3.91–5.21); 7.21x10^-95^ | | (11) | |  |
|  | GWAS (stage I) | European-Americans | 542 | 542 | 14.0 | 9.0 | 1.641 (1.254-2.148); 3.12x10^-4^ | | (12) | |  |
|  | GWAS (Stage II) | European-Americans | 544 | 687 | 32.8 | 12.0 | 3.58 (2.91-4.40); <1.00x10^-8^ | | (12) | |  |
| 12 | GWAS (stage III) | European-Americans | 324 | 702 | 31.0 | 14.0 | 2.76 (2.20-3.45); <1.00x10^-8^ | | (12) | |  |
| 13 | Meta-analysis | European-Americans | 2650 | 5578 | 30.4 | 11.8 | 3.73 ( 2.86–4.86); <1.0×10^-8^ | | (13) | |  |
|  |  | East-Asians | 209 | 1323 | 3.3 | 0.8 | 4.33 (2.19–8.59); 2.6×10^-6^ | | (13) | |  |
|  |  | Total | 2859 | 6901 | 28.2 | 9.6 | 3.77 (2.94–4.84); <1.0×10^-8^ | | (13) | |  |

Legend: MAF: Minor allele frequency; NHW: Non-Hispanic white; GWAS: Genome wide association study and NR: Not reported. Note: the designation “European-Americans” is used in the table because of brevity instead of more appropriate designation of this population as “U.S. population of European descent”(14).

**Table S2:** Genotype distribution of 26 variants studied, their genotype/ allele frequencies and carriage rate in Czech healthy controls (HC*), IPF cases from four European (CZ: Czechs, GE: Germans, GR: Greek and FR: French) and the combined IPF populations.

| S.No. | Gene variants | Genotype | **Genotype distribution** | | | | | | | | | | | | **Genotype frequency** | | | | | |  | **Allele frequency** | | | | | | **Carriage rate** | | | | | |
| --- | --- | --- | --- | --- | --- | --- | --- | --- | --- | --- | --- | --- | --- | --- | --- | --- | --- | --- | --- | --- | --- | --- | --- | --- | --- | --- | --- | --- | --- | --- | --- | --- | --- |
|  |  |  | **HC* n=96** | p | CZ n=41 | p | GE  n=33 | p | GR n=40 | p | FR n=51 | p | Total IPF n=165 | p | **HC*** | CZ | GE | GR | FR | Total IPF | Allele | **HC*** | CZ | GE | GR | FR | Total IPF | **HC*** | CZ | GE | GR | FR | Total IPF |
| 1 | IL-1 α | CC | 39 | 0.86 | 21 | 0.69 | 15 | 0.68 | 25 | 0.18 | 27 | 0.31 | 88 | 0.56 | 0.41 | 0.51 | 0.45 | 0.63 | 0.53 | 0.53 | C | 0.64 | 0.73 | 0.70 | 0.76 | 0.71 | 0.72 | 0.88 | 0.95 | 0.94 | 0.90 | 0.88 | 0.92 |
|  | rs1800587 | CT | 45 |  | 18 |  | 16 |  | 11 |  | 18 |  | 63 |  | 0.47 | 0.44 | 0.48 | 0.28 | 0.35 | 0.38 | T | 0.36 | 0.27 | 0.30 | 0.24 | 0.29 | 0.28 | 0.59 | 0.49 | 0.55 | 0.38 | 0.47 | 0.47 |
|  |  | TT | 12 |  | 2 |  | 2 |  | 4 |  | 6 |  | 14 |  | 0.13 | 0.05 | 0.06 | 0.10 | 0.12 | 0.08 |  |  |  |  |  |  |  |  |  |  |  |  |  |
| 2 | IL-1 β | GG | 46 | 0.35 | 18 | 0.51 | 17 | 1.00 | 18 | 1.00 | 22 | 0.76 | 75 | 0.73 | 0.48 | 0.44 | 0.52 | 0.45 | 0.43 | 0.45 | G | 0.68 | 0.65 | 0.73 | 0.68 | 0.65 | 0.67 | 0.88 | 0.85 | 0.94 | 0.90 | 0.86 | 0.88 |
|  | rs16944 | GA | 38 |  | 17 |  | 14 |  | 18 |  | 22 |  | 71 |  | 0.40 | 0.41 | 0.42 | 0.45 | 0.43 | 0.43 | A | 0.32 | 0.35 | 0.27 | 0.33 | 0.35 | 0.33 | 0.52 | 0.56 | 0.48 | 0.55 | 0.57 | 0.55 |
|  |  | AA | 12 |  | 6 |  | 2 |  | 4 |  | 7 |  | 19 |  | 0.13 | 0.15 | 0.06 | 0.10 | 0.14 | 0.12 |  |  |  |  |  |  |  |  |  |  |  |  |  |
| 3 | IL-1 β | CC | 56 | 0.49 | 21 | 0.24 | 15 | 0.68 | 27 | 0.60 | 28 | 1.00 | 91 | 0.53 | 0.58 | 0.51 | 0.45 | 0.68 | 0.55 | 0.55 | C | 0.76 | 0.74 | 0.70 | 0.81 | 0.75 | 0.75 | 0.93 | 0.98 | 0.94 | 0.95 | 0.94 | 0.95 |
|  | rs1143634 | CT | 33 |  | 19 |  | 16 |  | 11 |  | 20 |  | 66 |  | 0.34 | 0.46 | 0.48 | 0.28 | 0.39 | 0.40 | T | 0.24 | 0.26 | 0.30 | 0.19 | 0.25 | 0.25 | 0.42 | 0.49 | 0.55 | 0.33 | 0.45 | 0.45 |
|  |  | TT | 7 |  | 1 |  | 2 |  | 2 |  | 3 |  | 8 |  | 0.07 | 0.02 | 0.06 | 0.05 | 0.06 | 0.05 |  |  |  |  |  |  |  |  |  |  |  |  |  |
| 4 | PRKCE | GG | 61 | 0.11 | 19 | 0.72 | 21 | 0.61 | 24 | 0.09 | 42 | 0.39 | 106 | 0.02 | 0.64 | 0.48 | 0.64 | 0.60 | 0.82 | 0.65 | G | 0.78 | 0.68 | 0.79 | 0.74 | 0.90 | 0.78 | 0.93 | 0.88 | 0.94 | 0.88 | 0.98 | 0.92 |
|  | rs628877 | GT | 27 |  | 16 |  | 10 |  | 11 |  | 8 |  | 45 |  | 0.28 | 0.40 | 0.30 | 0.28 | 0.16 | 0.27 | T | 0.22 | 0.33 | 0.21 | 0.26 | 0.10 | 0.22 | 0.36 | 0.53 | 0.36 | 0.40 | 0.18 | 0.35 |
|  |  | TT | 7 |  | 5 |  | 2 |  | 5 |  | 1 |  | 13 |  | 0.07 | 0.13 | 0.06 | 0.13 | 0.02 | 0.08 |  |  |  |  |  |  |  |  |  |  |  |  |  |
| 5 | LRRC34 | TT | 45 | 0.23 | 21 | 0.69 | 17 | 1.00 | 22 | 1.00 | 19 | 0.38 | 79 | 0.46 | 0.47 | 0.51 | 0.52 | 0.55 | 0.37 | 0.48 | T | 0.70 | 0.73 | 0.71 | 0.75 | 0.64 | 0.70 | 0.94 | 0.95 | 0.91 | 0.95 | 0.90 | 0.93 |
|  | rs6793295 | TC | 45 |  | 18 |  | 13 |  | 16 |  | 27 |  | 74 |  | 0.47 | 0.44 | 0.39 | 0.40 | 0.53 | 0.45 | C | 0.30 | 0.27 | 0.29 | 0.25 | 0.36 | 0.30 | 0.53 | 0.49 | 0.48 | 0.45 | 0.63 | 0.52 |
|  |  | CC | 6 |  | 2 |  | 3 |  | 2 |  | 5 |  | 12 |  | 0.06 | 0.05 | 0.09 | 0.05 | 0.10 | 0.07 |  |  |  |  |  |  |  |  |  |  |  |  |  |
| 6 | TF | GG | 81 | 0.41 | 32 | 1.00 | 20 | 0.31 | 31 | 0.13 | 35 | 0.33 | 118 | 0.53 | 0.84 | 0.78 | 0.61 | 0.78 | 0.69 | 0.72 | G | 0.92 | 0.89 | 0.80 | 0.86 | 0.84 | 0.85 | 1.00 | 1.00 | 1.00 | 0.95 | 1.00 | 0.99 |
|  | rs1799899 | GA | 15 |  | 9 |  | 13 |  | 7 |  | 16 |  | 45 |  | 0.16 | 0.22 | 0.39 | 0.18 | 0.31 | 0.27 | A | 0.08 | 0.11 | 0.20 | 0.14 | 0.16 | 0.15 | 0.16 | 0.22 | 0.39 | 0.23 | 0.31 | 0.28 |
|  |  | AA | 0 |  | 0 |  | 0 |  | 2 |  | 0 |  | 2 |  | 0.00 | 0.00 | 0.00 | 0.05 | 0.00 | 0.01 |  |  |  |  |  |  |  |  |  |  |  |  |  |
| 7 | IL-8 | AA | 19 | 0.5 | 13 | 0.35 | 9 | 0.73 | 12 | 0.22 | 13 | 0.78 | 47 | 0.12 | 0.20 | 0.32 | 0.27 | 0.30 | 0.25 | 0.28 | A | 0.46 | 0.52 | 0.50 | 0.50 | 0.49 | 0.50 | 0.73 | 0.73 | 0.73 | 0.70 | 0.73 | 0.72 |
|  | rs4073 | AT | 51 |  | 17 |  | 15 |  | 16 |  | 24 |  | 72 |  | 0.53 | 0.41 | 0.45 | 0.40 | 0.47 | 0.44 | T | 0.54 | 0.48 | 0.50 | 0.50 | 0.51 | 0.50 | 0.80 | 0.68 | 0.73 | 0.70 | 0.75 | 0.72 |
|  |  | TT | 26 |  | 11 |  | 9 |  | 12 |  | 14 |  | 46 |  | 0.27 | 0.27 | 0.27 | 0.30 | 0.27 | 0.28 |  |  |  |  |  |  |  |  |  |  |  |  |  |
| 8 | FAM13A | TT | 53 | 0.89 | 25 | 0.09 | 21 | 0.61 | 21 | 0.70 | 22 | 1.00 | 89 | 0.56 | 0.55 | 0.61 | 0.64 | 0.53 | 0.43 | 0.54 | T | 0.74 | 0.74 | 0.79 | 0.74 | 0.67 | 0.73 | 0.94 | 0.88 | 0.94 | 0.95 | 0.90 | 0.92 |
|  | rs2609255 | GT | 37 |  | 11 |  | 10 |  | 17 |  | 24 |  | 62 |  | 0.39 | 0.27 | 0.30 | 0.43 | 0.47 | 0.38 | G | 0.26 | 0.26 | 0.21 | 0.26 | 0.33 | 0.27 | 0.45 | 0.39 | 0.36 | 0.48 | 0.57 | 0.46 |
|  |  | GG | 6 |  | 5 |  | 2 |  | 2 |  | 5 |  | 14 |  | 0.06 | 0.12 | 0.06 | 0.05 | 0.10 | 0.08 |  |  |  |  |  |  |  |  |  |  |  |  |  |
| 9 | TLR3 | GG | 41 | 0.88 | 18 | 1.00 | 21 | 0.06 | 20 | 0.69 | 28 | 0.48 | 87 | 0.45 | 0.43 | 0.44 | 0.64 | 0.50 | 0.55 | 0.53 | A | 0.66 | 0.67 | 0.76 | 0.73 | 0.73 | 0.72 | 0.89 | 0.90 | 0.88 | 0.95 | 0.90 | 0.91 |
|  | rs3775291 | GA | 44 |  | 19 |  | 8 |  | 18 |  | 18 |  | 63 |  | 0.46 | 0.46 | 0.24 | 0.45 | 0.35 | 0.38 | G | 0.34 | 0.33 | 0.24 | 0.28 | 0.27 | 0.28 | 0.57 | 0.56 | 0.36 | 0.50 | 0.45 | 0.47 |
|  |  | AA | 11 |  | 4 |  | 4 |  | 2 |  | 5 |  | 15 |  | 0.11 | 0.10 | 0.12 | 0.05 | 0.10 | 0.09 |  |  |  |  |  |  |  |  |  |  |  |  |  |
| 10 | TERT | TT | 29 | 0.91 | 13 | 0.75 | 11 | 1.00 | 6 | 0.75 | 11 | 0.41 | 41 | 1.00 | 0.30 | 0.32 | 0.33 | 0.15 | 0.22 | 0.25 | T | 0.55 | 0.55 | 0.59 | 0.38 | 0.50 | 0.50 | 0.80 | 0.78 | 0.85 | 0.60 | 0.78 | 0.75 |
|  | rs2736100 | GT | 48 |  | 19 |  | 17 |  | 18 |  | 29 |  | 83 |  | 0.50 | 0.46 | 0.52 | 0.45 | 0.57 | 0.50 | G | 0.45 | 0.45 | 0.41 | 0.63 | 0.50 | 0.50 | 0.70 | 0.68 | 0.67 | 0.85 | 0.78 | 0.75 |
|  |  | GG | 19 |  | 9 |  | 5 |  | 16 |  | 11 |  | 41 |  | 0.20 | 0.22 | 0.15 | 0.40 | 0.22 | 0.25 |  |  |  |  |  |  |  |  |  |  |  |  |  |
| 11 | IL-13 | CC | 49 | 0.68 | 23 | 0.16 | 20 | 1.00 | 20 | 0.45 | 29 | 0.71 | 92 | 1.00 | 0.51 | 0.58 | 0.61 | 0.51 | 0.57 | 0.56 | C | 0.71 | 0.79 | 0.79 | 0.69 | 0.75 | 0.75 | 0.91 | 1.00 | 0.97 | 0.87 | 0.92 | 0.94 |
|  | rs1800925 | CT | 38 |  | 17 |  | 12 |  | 14 |  | 18 |  | 61 |  | 0.40 | 0.43 | 0.36 | 0.36 | 0.35 | 0.37 | T | 0.29 | 0.21 | 0.21 | 0.31 | 0.25 | 0.25 | 0.49 | 0.43 | 0.39 | 0.49 | 0.43 | 0.44 |
|  |  | TT | 9 |  | 0 |  | 1 |  | 5 |  | 4 |  | 10 |  | 0.09 | 0.00 | 0.03 | 0.13 | 0.08 | 0.06 |  |  |  |  |  |  |  |  |  |  |  |  |  |
| 12 | IL-4 | TT | 80 | 0.44 | 38 | 1.00 | 31 | 0.05 | 30 | 1.00 | 42 | 1.00 | 141 | 1.00 | 0.85 | 0.93 | 0.94 | 0.75 | 0.82 | 0.85 | T | 0.93 | 0.96 | 0.95 | 0.88 | 0.91 | 0.92 | 1.00 | 1.00 | 0.97 | 1.00 | 1.00 | 0.99 |
|  | rs2243248 | GT | 14 |  | 3 |  | 1 |  | 10 |  | 9 |  | 23 |  | 0.15 | 0.07 | 0.03 | 0.25 | 0.18 | 0.14 | G | 0.07 | 0.04 | 0.05 | 0.13 | 0.09 | 0.08 | 0.15 | 0.07 | 0.06 | 0.25 | 0.18 | 0.15 |
|  |  | GG | 0 |  | 0 |  | 1 |  | 0 |  | 0 |  | 1 |  | 0.00 | 0.00 | 0.03 | 0.00 | 0.00 | 0.01 |  |  |  |  |  |  |  |  |  |  |  |  |  |
| 13 | IL-4 | CC | 64 | 0.24 | 33 | 0.01 | 25 | 0.13 | 32 | 0.09 | 38 | 0.06 | 128 | 1.2x10^-4^ | 0.68 | 0.80 | 0.76 | 0.80 | 0.76 | 0.78 | C | 0.81 | 0.87 | 0.85 | 0.88 | 0.85 | 0.86 | 0.95 | 0.93 | 0.94 | 0.95 | 0.94 | 0.94 |
|  | rs2243250 | CT | 25 |  | 5 |  | 6 |  | 6 |  | 9 |  | 26 |  | 0.27 | 0.12 | 0.18 | 0.15 | 0.18 | 0.16 | T | 0.19 | 0.13 | 0.15 | 0.13 | 0.15 | 0.14 | 0.32 | 0.20 | 0.24 | 0.20 | 0.24 | 0.22 |
|  |  | TT | 5 |  | 3 |  | 2 |  | 2 |  | 3 |  | 10 |  | 0.05 | 0.07 | 0.06 | 0.05 | 0.06 | 0.06 |  |  |  |  |  |  |  |  |  |  |  |  |  |
| 14 | IL-4 | CC | 66 | 0.91 | 32 | 0.02 | 24 | 1.00 | 32 | 1.00 | 34 | 1.00 | 122 | 0.35 | 0.69 | 0.78 | 0.73 | 0.80 | 0.67 | 0.74 | C | 0.83 | 0.85 | 0.86 | 0.90 | 0.81 | 0.85 | 0.97 | 0.93 | 1.00 | 1.00 | 0.96 | 0.97 |
|  | rs2070874 | CT | 27 |  | 6 |  | 9 |  | 8 |  | 15 |  | 38 |  | 0.28 | 0.15 | 0.27 | 0.20 | 0.29 | 0.23 | T | 0.17 | 0.15 | 0.14 | 0.10 | 0.19 | 0.15 | 0.31 | 0.22 | 0.27 | 0.20 | 0.33 | 0.26 |
|  |  | TT | 3 |  | 3 |  | 0 |  | 0 |  | 2 |  | 5 |  | 0.03 | 0.07 | 0.00 | 0.00 | 0.04 | 0.03 |  |  |  |  |  |  |  |  |  |  |  |  |  |
| 15 | CDKN1A | TT | 36 | 0.71 | 12 | 0.52 | 14 | 0.43 | 15 | 0.51 | 20 | 0.77 | 61 | 0.87 | 0.38 | 0.29 | 0.42 | 0.38 | 0.39 | 0.37 | T | 0.62 | 0.57 | 0.68 | 0.59 | 0.62 | 0.61 | 0.86 | 0.85 | 0.94 | 0.80 | 0.84 | 0.85 |
|  | rs733590 | CT | 47 |  | 23 |  | 17 |  | 17 |  | 23 |  | 80 |  | 0.49 | 0.56 | 0.52 | 0.43 | 0.45 | 0.48 | C | 0.38 | 0.43 | 0.32 | 0.41 | 0.38 | 0.39 | 0.63 | 0.71 | 0.58 | 0.63 | 0.61 | 0.63 |
|  |  | CC | 13 |  | 6 |  | 2 |  | 8 |  | 8 |  | 24 |  | 0.14 | 0.15 | 0.06 | 0.20 | 0.16 | 0.15 |  |  |  |  |  |  |  |  |  |  |  |  |  |
| 16 | OBFC1 | GG | 33 | 0.7 | 7 | 0.53 | 5 | 0.48 | 15 | 0.20 | 13 | 0.78 | 40 | 0.88 | 0.34 | 0.17 | 0.15 | 0.38 | 0.25 | 0.24 | G | 0.58 | 0.45 | 0.44 | 0.56 | 0.49 | 0.49 | 0.81 | 0.73 | 0.73 | 0.75 | 0.73 | 0.73 |
|  | rs11191865 | AG | 45 |  | 23 |  | 19 |  | 15 |  | 24 |  | 81 |  | 0.47 | 0.56 | 0.58 | 0.38 | 0.47 | 0.49 | A | 0.42 | 0.55 | 0.56 | 0.44 | 0.51 | 0.51 | 0.66 | 0.83 | 0.85 | 0.63 | 0.75 | 0.76 |
|  |  | AA | 18 |  | 11 |  | 9 |  | 10 |  | 14 |  | 44 |  | 0.19 | 0.27 | 0.27 | 0.25 | 0.27 | 0.27 |  |  |  |  |  |  |  |  |  |  |  |  |  |
| 17 | MUC2 | GG | 41 | 0.16 | 15 | 1.00 | 3 | 0.07 | 9 | 0.75 | 11 | 1.00 | 38 | 0.75 | 0.43 | 0.37 | 0.09 | 0.23 | 0.22 | 0.23 | G | 0.68 | 0.60 | 0.42 | 0.46 | 0.47 | 0.49 | 0.93 | 0.83 | 0.76 | 0.69 | 0.73 | 0.75 |
|  | rs7934606 | AG | 48 |  | 19 |  | 22 |  | 18 |  | 26 |  | 85 |  | 0.50 | 0.46 | 0.67 | 0.46 | 0.51 | 0.52 | A | 0.32 | 0.40 | 0.58 | 0.54 | 0.53 | 0.51 | 0.57 | 0.63 | 0.91 | 0.77 | 0.78 | 0.77 |
|  |  | AA | 7 |  | 7 |  | 8 |  | 12 |  | 14 |  | 41 |  | 0.07 | 0.17 | 0.24 | 0.31 | 0.27 | 0.25 |  |  |  |  |  |  |  |  |  |  |  |  |  |
| 18 | MUC5B | GG | 80 | 0.16 | 21 | 1.00 | 12 | 0.06 | 16 | 0.47 | 18 | 1.00 | 67 | 0.86 | 0.83 | 0.51 | 0.36 | 0.44 | 0.35 | 0.42 | G | 0.91 | 0.72 | 0.67 | 0.64 | 0.59 | 0.65 | 0.98 | 0.93 | 0.97 | 0.83 | 0.82 | 0.88 |
|  | rs35705950 | GT | 14 |  | 17 |  | 20 |  | 14 |  | 24 |  | 75 |  | 0.15 | 0.41 | 0.61 | 0.39 | 0.47 | 0.47 | T | 0.09 | 0.28 | 0.33 | 0.36 | 0.41 | 0.35 | 0.17 | 0.49 | 0.64 | 0.56 | 0.65 | 0.58 |
|  |  | TT | 2 |  | 3 |  | 1 |  | 6 |  | 9 |  | 19 |  | 0.02 | 0.07 | 0.03 | 0.17 | 0.18 | 0.12 |  |  |  |  |  |  |  |  |  |  |  |  |  |
| 19 | ATP11A | GG | 48 | 0.79 | 18 | 0.72 | 22 | 1.00 | 20 | 0.72 | 34 | 1.00 | 94 | 1.00 | 0.50 | 0.44 | 0.67 | 0.50 | 0.67 | 0.57 | G | 0.70 | 0.68 | 0.82 | 0.70 | 0.82 | 0.76 | 0.91 | 0.93 | 0.97 | 0.90 | 0.98 | 0.95 |
|  | rs1278769 | GA | 39 |  | 20 |  | 10 |  | 16 |  | 16 |  | 62 |  | 0.41 | 0.49 | 0.30 | 0.40 | 0.31 | 0.38 | A | 0.30 | 0.32 | 0.18 | 0.30 | 0.18 | 0.24 | 0.50 | 0.56 | 0.33 | 0.50 | 0.33 | 0.43 |
|  |  | AA | 9 |  | 3 |  | 1 |  | 4 |  | 1 |  | 9 |  | 0.09 | 0.07 | 0.03 | 0.10 | 0.02 | 0.05 |  |  |  |  |  |  |  |  |  |  |  |  |  |
| 20 | IL-4R α | AA | 68 | 0.04 | 26 | 0.37 | 18 | 0.67 | 28 | 0.03 | 29 | 0.08 | 101 | 0.01 | 0.71 | 0.63 | 0.55 | 0.70 | 0.57 | 0.61 | A | 0.82 | 0.78 | 0.73 | 0.80 | 0.72 | 0.75 | 0.94 | 0.93 | 0.91 | 0.90 | 0.86 | 0.90 |
|  | rs1801275 | AG | 22 |  | 12 |  | 12 |  | 8 |  | 15 |  | 47 |  | 0.23 | 0.29 | 0.36 | 0.20 | 0.29 | 0.28 | G | 0.18 | 0.22 | 0.27 | 0.20 | 0.28 | 0.25 | 0.29 | 0.37 | 0.45 | 0.30 | 0.43 | 0.39 |
|  |  | GG | 6 |  | 3 |  | 3 |  | 4 |  | 7 |  | 17 |  | 0.06 | 0.07 | 0.09 | 0.10 | 0.14 | 0.10 |  |  |  |  |  |  |  |  |  |  |  |  |  |
| 21 | TP53 | AA | 81 | 0.41 | 28 | 0.59 | 25 | 1.00 | 37 | 1.00 | 46 | 0.14 | 136 | 0.18 | 0.84 | 0.68 | 0.76 | 0.93 | 0.90 | 0.82 | A | 0.92 | 0.82 | 0.88 | 0.96 | 0.94 | 0.90 | 1.00 | 0.95 | 1.00 | 1.00 | 0.98 | 0.98 |
|  | rs12951053 | CA | 15 |  | 11 |  | 8 |  | 3 |  | 4 |  | 26 |  | 0.16 | 0.27 | 0.24 | 0.08 | 0.08 | 0.16 | C | 0.08 | 0.18 | 0.12 | 0.04 | 0.06 | 0.10 | 0.16 | 0.32 | 0.24 | 0.08 | 0.10 | 0.18 |
|  |  | CC | 0 |  | 2 |  | 0 |  | 0 |  | 1 |  | 3 |  | 0.00 | 0.05 | 0.00 | 0.00 | 0.02 | 0.02 |  |  |  |  |  |  |  |  |  |  |  |  |  |
| 22 | TP53 | CC | 81 | 0.41 | 29 | 0.31 | 26 | 1.00 | 34 | 0.25 | 40 | 0.52 | 129 | 0.26 | 0.84 | 0.71 | 0.79 | 0.85 | 0.78 | 0.78 | C | 0.92 | 0.83 | 0.89 | 0.91 | 0.88 | 0.88 | 1.00 | 0.95 | 1.00 | 0.98 | 0.98 | 0.98 |
|  | rs12602273 | GC | 15 |  | 10 |  | 7 |  | 5 |  | 10 |  | 32 |  | 0.16 | 0.24 | 0.21 | 0.13 | 0.20 | 0.19 | G | 0.08 | 0.17 | 0.11 | 0.09 | 0.12 | 0.12 | 0.16 | 0.29 | 0.21 | 0.15 | 0.22 | 0.22 |
|  |  | GG | 0 |  | 2 |  | 0 |  | 1 |  | 1 |  | 4 |  | 0.00 | 0.05 | 0.00 | 0.03 | 0.02 | 0.02 |  |  |  |  |  |  |  |  |  |  |  |  |  |
| 23 | MAPT | GG | 67 | 0.81 | 30 | 0.24 | 22 | 0.12 | 31 | 0.47 | 18 | 0.03 | 101 | 0.82 | 0.70 | 0.73 | 0.67 | 0.78 | 0.36 | 0.62 | G | 0.83 | 0.84 | 0.79 | 0.88 | 0.66 | 0.78 | 0.97 | 0.95 | 0.91 | 0.98 | 0.96 | 0.95 |
|  | rs1981997 | GA | 26 |  | 9 |  | 8 |  | 8 |  | 30 |  | 55 |  | 0.27 | 0.22 | 0.24 | 0.20 | 0.60 | 0.34 | A | 0.17 | 0.16 | 0.21 | 0.13 | 0.34 | 0.22 | 0.30 | 0.27 | 0.33 | 0.23 | 0.64 | 0.38 |
|  |  | AA | 3 |  | 2 |  | 3 |  | 1 |  | 2 |  | 8 |  | 0.03 | 0.05 | 0.09 | 0.03 | 0.04 | 0.05 |  |  |  |  |  |  |  |  |  |  |  |  |  |
| 24 | ACE | TT | 36 | 0.51 | 23 | 1.00 | 12 | 1.00 | 13 | 0.51 | 15 | 0.09 | 63 | 0.18 | 0.38 | 0.56 | 0.36 | 0.33 | 0.29 | 0.38 | T | 0.63 | 0.76 | 0.61 | 0.60 | 0.60 | 0.64 | 0.88 | 0.95 | 0.85 | 0.88 | 0.90 | 0.90 |
|  | rs4277405 | CT | 48 |  | 16 |  | 16 |  | 22 |  | 31 |  | 85 |  | 0.50 | 0.39 | 0.48 | 0.55 | 0.61 | 0.52 | C | 0.38 | 0.24 | 0.39 | 0.40 | 0.40 | 0.36 | 0.63 | 0.44 | 0.64 | 0.68 | 0.71 | 0.62 |
|  |  | CC | 12 |  | 2 |  | 5 |  | 5 |  | 5 |  | 17 |  | 0.13 | 0.05 | 0.15 | 0.13 | 0.10 | 0.10 |  |  |  |  |  |  |  |  |  |  |  |  |  |
| 25 | ACE | AA | 37 | 0.83 | 23 | 1.00 | 12 | 1.00 | 13 | 0.51 | 16 | 0.14 | 64 | 0.23 | 0.39 | 0.56 | 0.36 | 0.33 | 0.31 | 0.39 | A | 0.63 | 0.76 | 0.61 | 0.60 | 0.61 | 0.64 | 0.86 | 0.95 | 0.85 | 0.88 | 0.90 | 0.90 |
|  | rs4459609 | CA | 46 |  | 16 |  | 16 |  | 22 |  | 30 |  | 84 |  | 0.48 | 0.39 | 0.48 | 0.55 | 0.59 | 0.51 | C | 0.38 | 0.24 | 0.39 | 0.40 | 0.39 | 0.36 | 0.61 | 0.44 | 0.64 | 0.68 | 0.69 | 0.61 |
|  |  | CC | 13 |  | 2 |  | 5 |  | 5 |  | 5 |  | 17 |  | 0.14 | 0.05 | 0.15 | 0.13 | 0.10 | 0.10 |  |  |  |  |  |  |  |  |  |  |  |  |  |
| 26 | DPP9 | AA | 54 | 0.69 | 23 | 0.70 | 15 | 0.46 | 21 | 1.00 | 25 | 0.73 | 84 | 1.00 | 0.56 | 0.56 | 0.45 | 0.54 | 0.50 | 0.52 | A | 0.74 | 0.74 | 0.65 | 0.74 | 0.72 | 0.72 | 0.93 | 0.93 | 0.85 | 0.95 | 0.94 | 0.92 |
|  | rs12610495 | GA | 35 |  | 15 |  | 13 |  | 16 |  | 22 |  | 66 |  | 0.36 | 0.37 | 0.39 | 0.41 | 0.44 | 0.40 | G | 0.26 | 0.26 | 0.35 | 0.26 | 0.28 | 0.28 | 0.44 | 0.44 | 0.55 | 0.46 | 0.50 | 0.48 |
|  |  | GG | 7 |  | 3 |  | 5 |  | 2 |  | 3 |  | 13 |  | 0.07 | 0.07 | 0.15 | 0.05 | 0.06 | 0.08 |  |  |  |  |  |  |  |  |  |  |  |  |  |

*ACE* (Angiotensin-converting enzyme); *ATP11A* (ATPase, class VI, type 11A); *CDKN1A* (cyclin-dependent kinase inhibitor 1A, the gene encoding p21); *DPP9* (Dipeptidyl-peptidase 9); *FAM13A* (Family with sequence similarity 13, member A); *IL* (Interleukins); *LRRC34* (Leucine rich repeat containing 34); *MAPT* (Microtubule-associated protein tau); *MUC2* (Mucin 2); *MUC5B* (Mucin 5B); *OBFC1* (Oligonucleotide/oligosaccharide-binding fold containing 1); *PKCE* (Protein kinase C, epsilon); *SFTP* (Surfactant protein); *SPPL2C* (signal peptide peptidase like 2C); *TERT* (Telomerase reverse transcriptase); *TF* (Transferrin); *TGF-β* (Transforming growth factor-β); *TLR3* (Toll like receptor); *TNF-a* (Tumour necrosis factor-α); *TP53* (tumour protein 53). **Healthy controls from our previous data report (15).*

**References:**

## 1. Seibold MA, Wise AL, Speer MC, Steele MP, Brown KK, Loyd JE, et al. A common MUC5B promoter polymorphism and pulmonary fibrosis. *N Engl J Med* (2011) 364:1503-12. doi:10.1056/NEJMoa1013660

## 2. Hunninghake GM, Hatabu H, Okajima Y, Gao W, Dupuis J, Latourelle JC, et al. MUC5B promoter polymorphism and interstitial lung abnormalities. *N Engl J Med* (2013) 368:2192-200. doi:10.1056/NEJMoa1216076

## 3. Peljto AL, Selman M, Kim DS, Murphy E, Tucker L, Pardo A, et al. The MUC5B promoter polymorphism is associated with idiopathic pulmonary fibrosis in a Mexican cohort but is rare among Asian ancestries. *Chest* (2015) 147:460-4. doi:10.1378/chest.14-0867

## 4. Zhang Y, Noth I, Garcia JG and Kaminski N A variant in the promoter of MUC5B and idiopathic pulmonary fibrosis. *N Engl J Med* (2011) 364:1576-7. doi:10.1056/NEJMc1013504

## 5. Horimasu Y, Ohshimo S, Bonella F, Tanaka S, Ishikawa N, Hattori N, et al. MUC5B promoter polymorphism in Japanese patients with idiopathic pulmonary fibrosis. *Respirology* (2015) 20:439-44. doi:10.1111/resp.12466

## 6. Wang C, Zhuang Y, Guo W, Cao L, Zhang H, Xu L, et al. Mucin 5B promoter polymorphism is associated with susceptibility to interstitial lung diseases in Chinese males. *PLoS One* (2014) 9:e104919. doi:10.1371/journal.pone.0104919

## 7. Wei R, Li C, Zhang M, Jones-Hall YL, Myers JL, Noth I, et al. Association between MUC5B and TERT polymorphisms and different interstitial lung disease phenotypes. *Transl Res* (2014) 163:494-502. doi:10.1016/j.trsl.2013.12.006

## 8. van der Vis JJ, Snetselaar R, Kazemier KM, Ten Klooster L, Grutters JC and van Moorsel CH Effect of Muc5b promoter polymorphism on disease predisposition and survival in idiopathic interstitial pneumonias. *Respirology* (2015). doi:10.1111/resp.12728

## 9. Borie R, Crestani B, Dieude P, Nunes H, Allanore Y, Kannengiesser C, et al. The MUC5B variant is associated with idiopathic pulmonary fibrosis but not with systemic sclerosis interstitial lung disease in the European Caucasian population. *PLoS One* (2013) 8:e70621. doi:10.1371/journal.pone.0070621

## 10. Stock CJ, Sato H, Fonseca C, Banya WA, Molyneaux PL, Adamali H, et al. Mucin 5B promoter polymorphism is associated with idiopathic pulmonary fibrosis but not with development of lung fibrosis in systemic sclerosis or sarcoidosis. *Thorax* (2013) 68:436-41. doi:10.1136/thoraxjnl-2012-201786

## 11. Fingerlin TE, Murphy E, Zhang W, Peljto AL, Brown KK, Steele MP, et al. Genome-wide association study identifies multiple susceptibility loci for pulmonary fibrosis. *Nat Genet* (2013) 45:613-20. doi:10.1038/ng.2609

## 12. Noth I, Zhang Y, Ma SF, Flores C, Barber M, Huang Y, et al. Genetic variants associated with idiopathic pulmonary fibrosis susceptibility and mortality: a genome-wide association study. *Lancet Respir Med* (2013) 1:309-17. doi:10.1016/S2213-2600(13)70045-6

## 13. Lee MG and Lee YH A meta-analysis examining the association between the MUC5B rs35705950 T/G polymorphism and susceptibility to idiopathic pulmonary fibrosis. *Inflamm Res* (2015) 64:463-70. doi:10.1007/s00011-015-0829-6

## 14. Sanchez-Mazas A, Vidan-Jeras B, Nunes JM, Fischer G, Little AM, Bekmane U, et al. Strategies to work with HLA data in human populations for histocompatibility, clinical transplantation, epidemiology and population genetics: HLA-NET methodological recommendations. *Int J Immunogenet* (2012) 39:459-72; quiz 73-6. doi:10.1111/j.1744-313X.2012.01113.x

15. Kishore A, Zizkova V, Kocourkova L and Petrek M A Dataset of 26 Candidate Gene and Pro-Inflammatory Cytokine Variants for Association Studies in Idiopathic Pulmonary Fibrosis: Frequency Distribution in Normal Czech Population. *Front Immunol* (2015) **6:**476. doi:10.3389/fimmu.2015.00476
