# Supplementary material for: Association Study for 26 Candidate Loci in Idiopathic Pulmonary Fibrosis Patients from Four European Populations
Source: Front Immunol. 2016 Jul 11;7:274. doi: 10.3389/fimmu.2016.00274 (PMC4939450; doi:10.3389/fimmu.2016.00274)
Supplement: Supplementary file 2 [file Image_1.PDF]

# *Supplementary Material*

# Association Study for 26 Candidate Loci in Idiopathic Pulmonary Fibrosis Patients from Four European Populations

**Amit Kishore[1], Veronika Žižková[1], Lenka Kocourková[1], Jana Petrkova[1], Evangelos Bouros[2], Hilario Nunes[3], Vladimíra Loštáková[4], Joachim Müller-Quernheim[5], Gernot Zissel[5], Vitezslav Kolek[4], Demosthenes Bouros[6], Dominique Valeyre[3], Martin Petrek [1,7]***

[1]Department of Pathological Physiology, Laboratory of Immunogenomics, Faculty of Medicine and Dentistry, Palacký University, Olomouc, Czech Republic

[2]Pharmacology Lab, Democritus University of Thrace and University Hospital Alexandroupolis, Athens, Greece

[3]Université Paris 13, COMUE Sorbonne Paris Cité, Bobigny - Paris, France

[4]Department of Respiratory Medicine, Faculty of Medicine and Dentistry, Palacký University, Olomouc, Czech Republic

[5]Department of Pneumology, Center for Medicine, Medical Center, University of Freiburg, Freiburg, Germany

[6]Academic Department of Pneumonology, Medical School, University of Athens, Hospital for Diseases of the Chest 'Sotiria' - Athens, Greece

[7]Institute of Molecular and Translational Medicine, Faculty of Medicine and Dentistry, Palacký University, Olomouc, Czech Republic

***Correspondence:** dr. M. Petrek, Department of Pathological Physiology, Faculty of Medicine and Dentistry, Palacky University, Olomouc, Hněvotínská str. 3, 77515, Czech Republic; E-mail: martin.petrek@fnol.cz

## Supplementary Figures

**Fig S1:** Interaction of genes with candidate loci for IPF in the same gene family (*MUC5B* and *MUC2*).

**Fig S2:** Transcriptional interaction of genes with candidate loci (*MUC2* and *TP53*) for IPF.

**Fig S3:** Interaction of genes with candidate loci for IPF in the same biosystem (*MUC5B*, *MUC2*, *TERT*, *TF*, *TP53*, *ACE*, *IL1A* and *ATP11A*).

**Supplementary Figures**

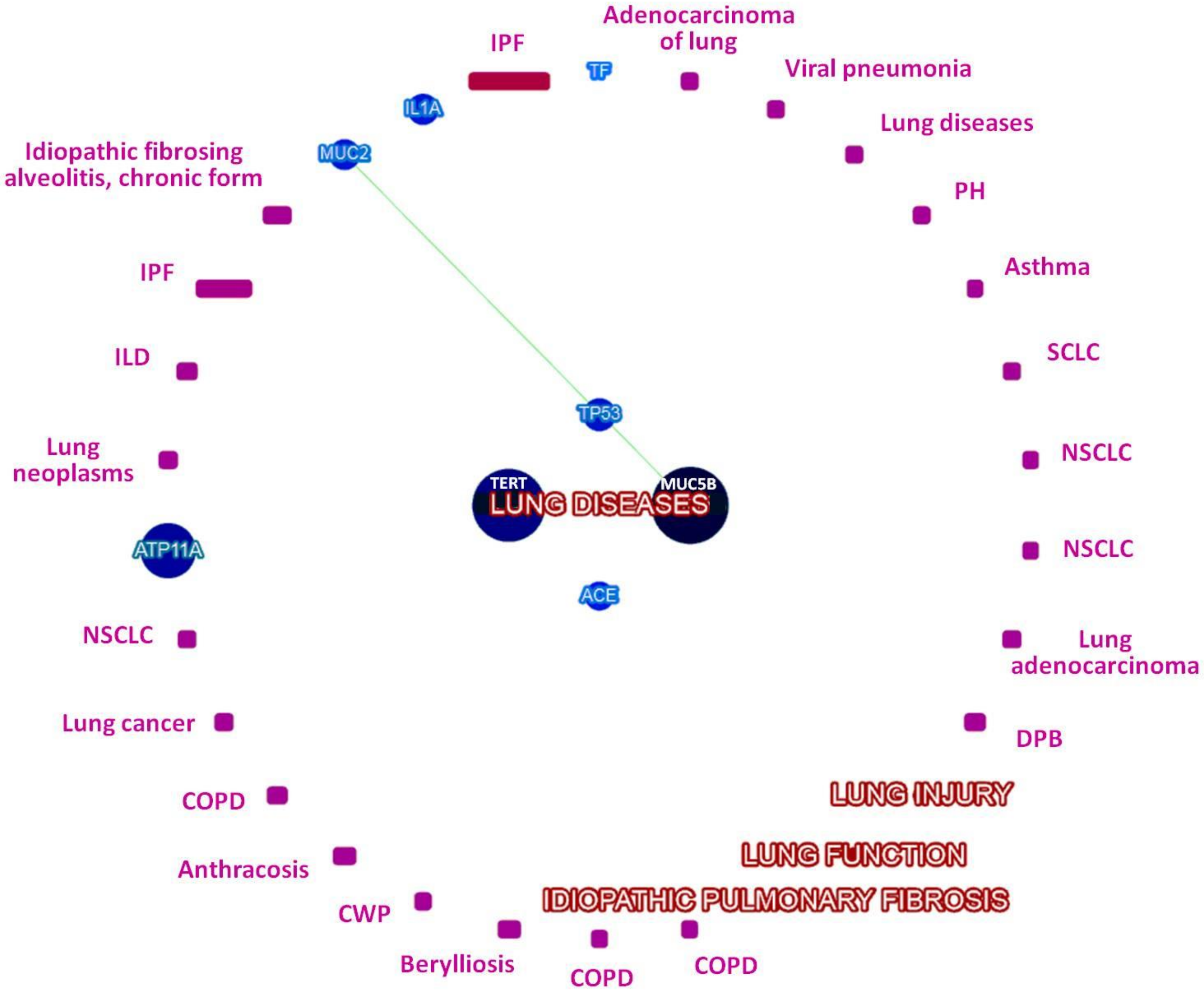


**Fig S1:** Interaction of genes with candidate loci for IPF in the same gene family (*MUC5B* and *MUC2*).

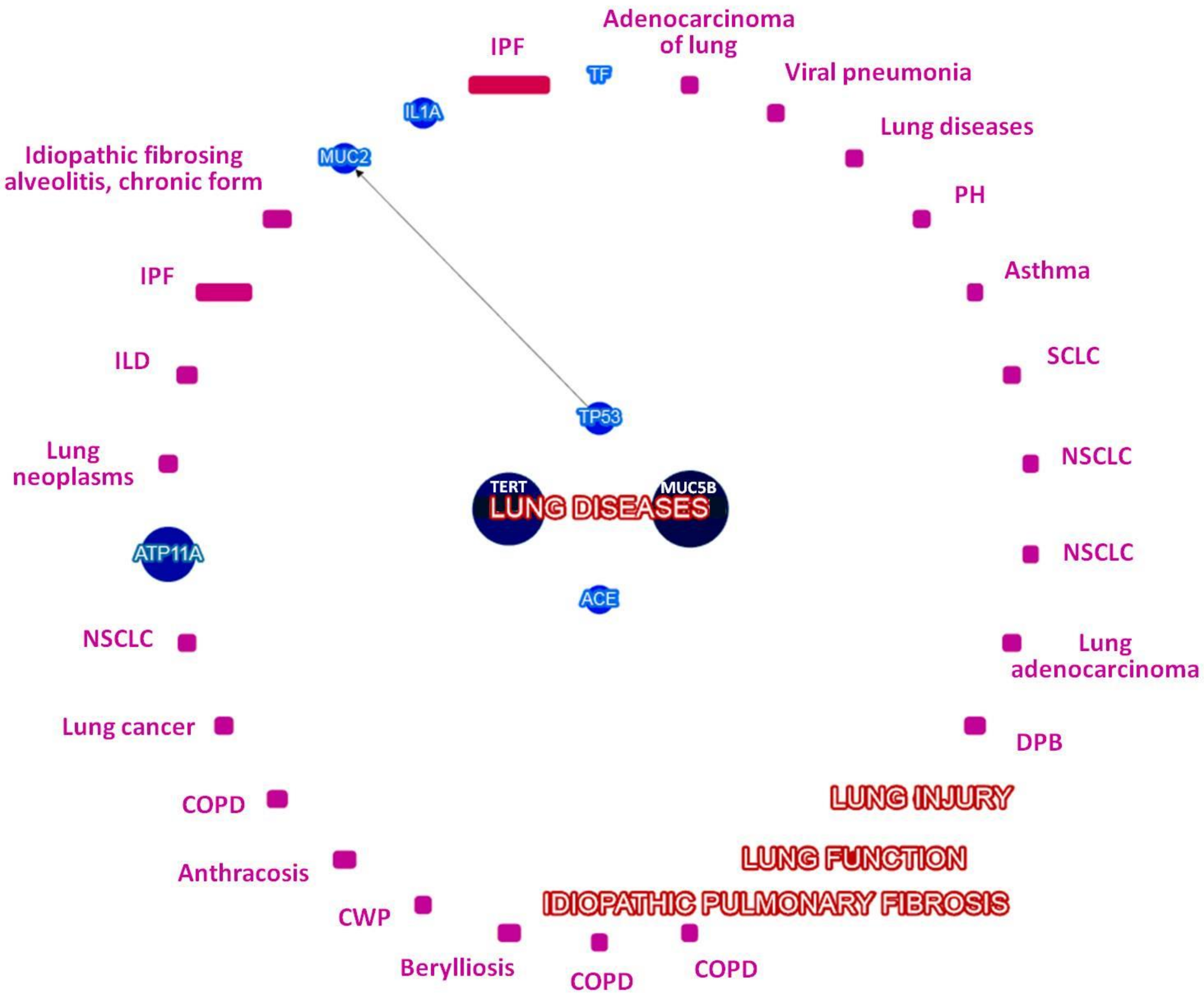


**Fig S2:** Transcriptional interaction of genes with candidate loci (*MUC2* and *TP53*) for IPF.

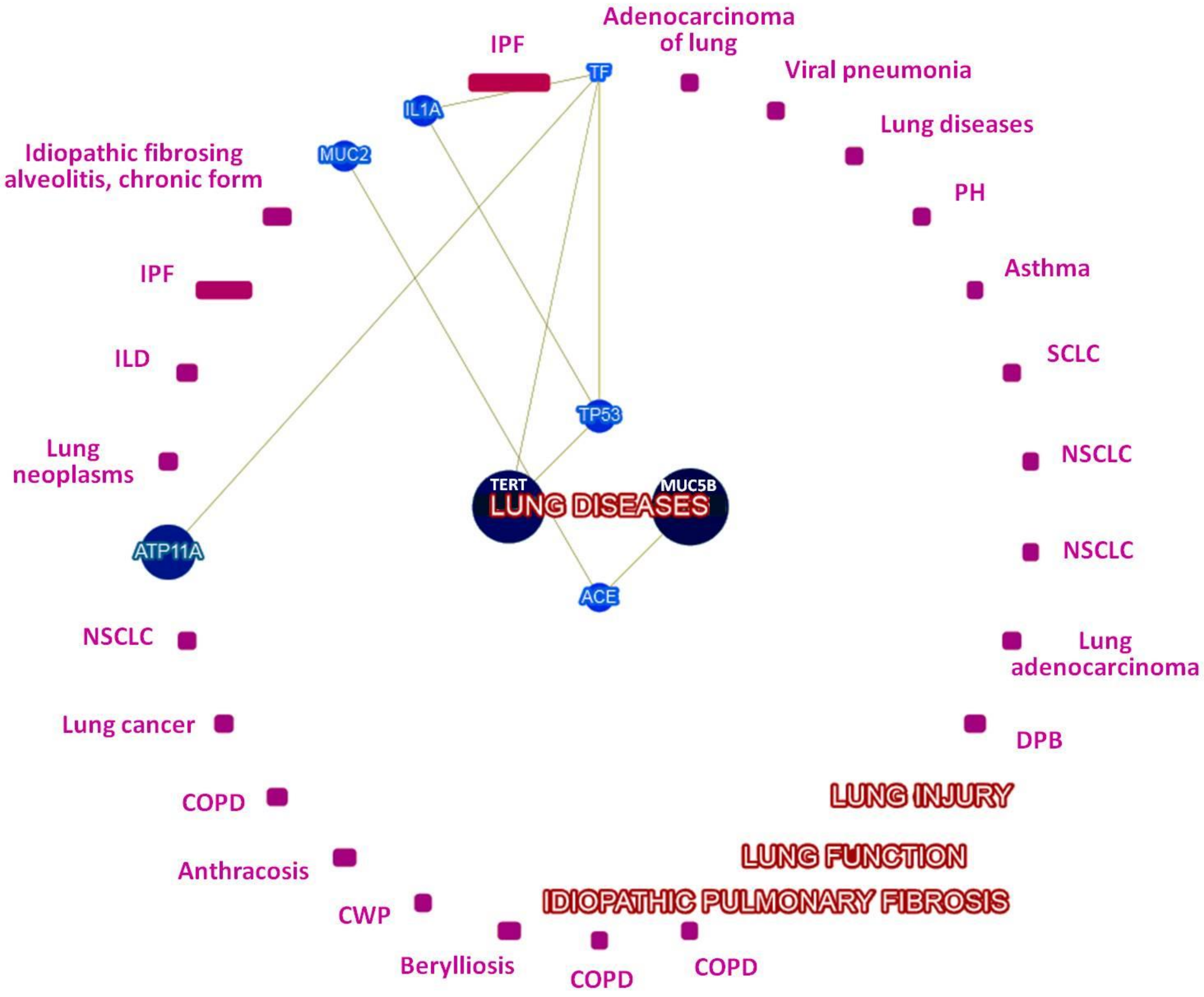


**Fig S3:** Interaction of genes with candidate loci for IPF in the same biosystem (*MUC5B*, *MUC2*, *TERT*, *TF*, *TP53*, *ACE*, *IL1A* and *ATP11A*).
